# Three-dimensional alignment changes of the shoulder girdle between the supine and standing positions

**DOI:** 10.1186/s13018-020-01934-w

**Published:** 2020-09-15

**Authors:** Noboru Matsumura, Yoshitake Yamada, Satoshi Oki, Yuki Yoshida, Yoichi Yokoyama, Minoru Yamada, Takeo Nagura, Masahiro Jinzaki

**Affiliations:** 1 Department of Orthopedic Surgery, Keio University School of Medicine, 35 Shinanomachi, Shinjuku-ku, Tokyo, 160-8582 Japan; 2 Department of Radiology, Keio University School of Medicine, 35 Shinanomachi, Shinjuku-ku, Tokyo, 160-8582 Japan

**Keywords:** Shoulder girdle, Upright computed tomography, Shoulder girdle alignment, Clavicle rotation, Scapula rotation, Scapulothoracic joint

## Abstract

**Background:**

Although humans spend most of their day in a standing or sitting position, it is difficult to accurately evaluate the alignment of the shoulder girdle during daily activity, and its alignment changes between positions. The purpose of this study was to evaluate the 3-dimensional alignment of the shoulder girdle in the supine and standing positions by computed tomography (CT) and to assess the alignment changes of the shoulder girdle between these two positions.

**Methods:**

CT scans of both shoulders of 100 healthy volunteers were prospectively taken in both supine and standing positions on the same day. The local 3-dimensional coordinate systems of the thorax, clavicle, and scapula were defined from the specific bony landmarks, and 3-dimensional angular rotations and positions of the clavicle and scapula were calculated. Differences in rotations and positions of the clavicle and scapula were evaluated between the supine and standing positions.

**Results:**

Compared with the supine position, the clavicle showed significantly less elevation and greater retraction, and the scapula showed significantly less upward rotation, anterior tilting, and internal rotation in the standing position. Compared with the supine position, the clavicle center was located more inferiorly, posteriorly, and laterally, and the scapula center was located more inferiorly, posteriorly, and medially in the standing position.

**Conclusions:**

The present study showed that angular rotations and positions of the clavicle and scapula change significantly with position due to the effect of gravity.

## Introduction

Humans perform their activities of daily living mainly in a standing or sitting position, but it is difficult to accurately evaluate the alignment of the shoulder girdle during daily activities. Plain radiographs can be taken in an upright position, but 3-dimensional anatomical alignment cannot be assessed on 2-dimensional projection images [1]. On the other hand, computed tomography (CT) scans have the advantage of assessing bones and joints three dimensionally, and they are now widely used to evaluate joint pathologies, but CT scans are usually taken in the supine position. Direct measurement [2] and measurement using electromagnetic tracking devices [3–6] require palpation for identification of the bony landmarks from outside of the human body, and these measurement methods cannot exclude the effect of the skin artifact. Though the anatomical alignment of the shoulder girdle can change with body position, its alignment changes between positions have not been evaluated.

A newly developed upright CT scanner, whose transverse 320 row-detector gantry includes a stand supporting the rotary section on either side with a linear motion rail and ball screw to move the scanner up and down, enables whole-torso cross-sectional scanning with a 3-dimensional acquisition in the standing position [7] and evaluation of the effect of gravity on the human body [8]. The purpose of this study was to evaluate the 3-dimensional alignment of the shoulder girdle in the supine position using a conventional CT scanner and in the standing position using a newly developed upright CT scanner and to assess the alignment changes of the girdle between these two positions. We hypothesized that angular rotations and positions of the shoulder girdle differ between the supine and standing positions due to the effect of gravity.

## Methods

### Participants

This study was approved by the Institutional Review Board of Keio University School of Medicine (reference study number 20160384), and written, informed consent was obtained from all participants. The inclusion criteria for this study were healthy volunteers without any past medical history, age ranging from 30 to 60 years, and who showed complete understanding of the details of their involvement and provided their informed consent to participate in this study. The exclusion criteria were shoulders with obvious degenerative changes in the sternoclavicular, acromioclavicular, and glenohumeral joints, and spinal scoliosis with Cobb’s angle greater than 10° [9] on the CT scans obtained. A total of 106 healthy volunteers were prospectively recruited by a volunteer recruitment company. Of these, 6 participants were excluded because CT showed asymptomatic spinal scoliosis. Thus, 200 shoulders from 100 healthy volunteers (60 females and 40 males) were included in the analysis. The participants’ mean (± standard deviation) age, height, and weight were 43.2 ± 8.0 years (range, 30–59 years), 171.4 ± 6.4 cm (range, 159.1–187.5 cm), and 68.1 ± 9.6 kg (range, 47.4–88.2 kg) in males, and 44.9 ± 8.5 years (range, 30–60 years), 157.0 ± 5.2 cm (range, 147.7–170.7 cm), and 53.9 ± 8.0 kg (range, 37.8–77.5 kg) in females, respectively.

**Fig. 1:**
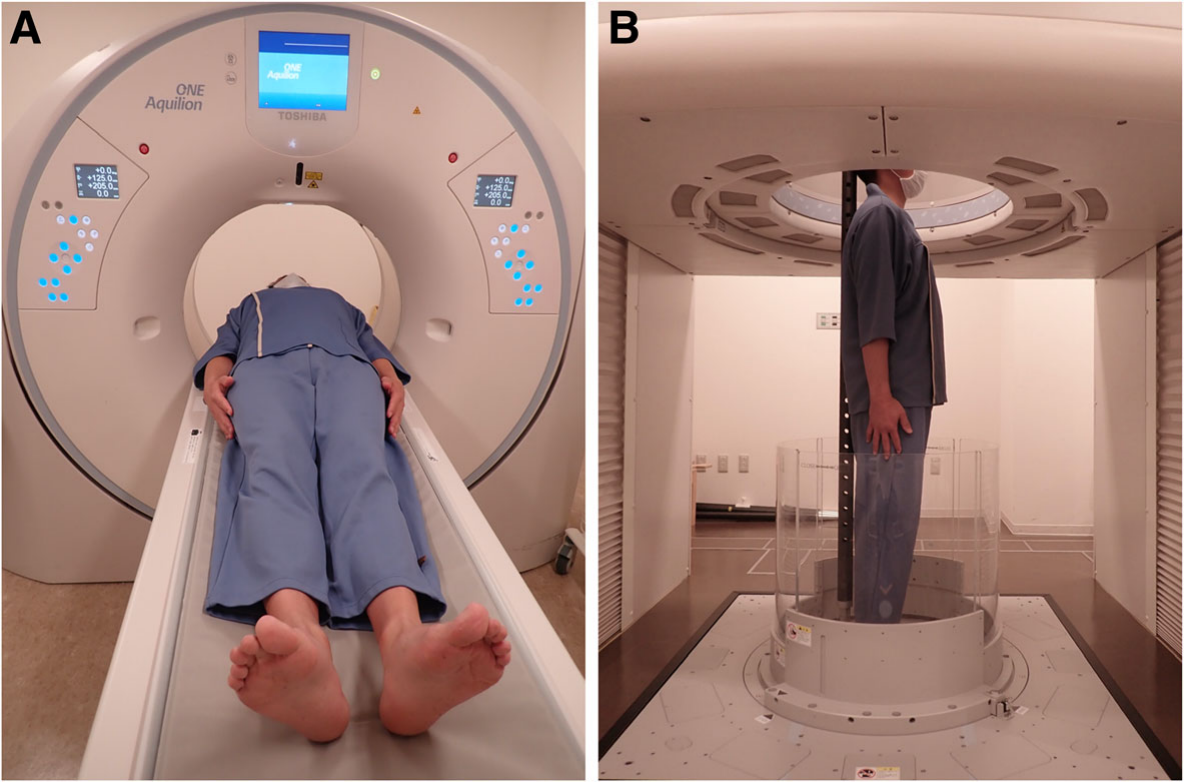
Computed tomography scanning in the supine and standing positions. **a** CT scans of both shoulders of the volunteers are taken in the supine position using a conventional 320-detector row CT scanner. **b** CT scans are taken in the standing position using an upright CT scanner, which has a transverse 320 row-detector gantry. A support pole lightly supports the back of the volunteer, and up-and-down movements of the transverse gantry enable cross-sectional scanning in the standing position

CT scans of both shoulders of the volunteers were taken in the supine position using a conventional 320-detector row CT scanner (Aquilion ONE; Canon Medical Systems Corporation, Otawara, Japan) and in the standing position using an upright 320-detector row CT scanner (prototype TSX-401R; Canon Medical Systems Corporation) on the same day. During CT scanning in the supine position, the volunteers were placed on the floor of the CT scanner with their arms at their sides (Fig. 1a). During upright CT scanning, the volunteers stood in the transverse gantry with the shoulders adducted and the arms held in the neutral position, and a support pole was placed behind them to stabilize the back (Fig. 1b). CT scans of both shoulders in the standing position were acquired during up-and-down movements of the transverse gantry [7]. The total effective dose of radiation exposure was tracked during all CT scanning and was controlled to not exceed 30 mSv. The image data were extracted in the obtained Digital Imaging and Communication in Medicine data format.

### Measurements of shoulder girdle alignment

Using OsiriX MD 11.0.0 software (Pixmeo, Geneva, Switzerland), the specific bony landmarks were identified on both supine and standing CT scans of 200 shoulders. According to the International Society of Biomechanics standardization proposal for the upper extremity, the 7th cervical vertebra (C7), 8th thoracic vertebra (T8), sternal notch (incisura jugularis; IJ), and xiphoid process (processus xiphoideus; PX) were digitized as the thoracic landmarks [10]. The sternoclavicular joint, which was defined as the most ventral point on the proximal end of the clavicle (SC), and the acromioclavicular joint, which was defined as the most dorsal point on the distal end of the clavicle (AC), were used for the clavicle, whereas the root of the scapular spine (trigonum spinae scapulae; TS), inferior angle (angulus inferior; AI), and posterolateral edge of the acromion (angulus acromialis; AA) were used for the scapula.

**Fig. 2:**
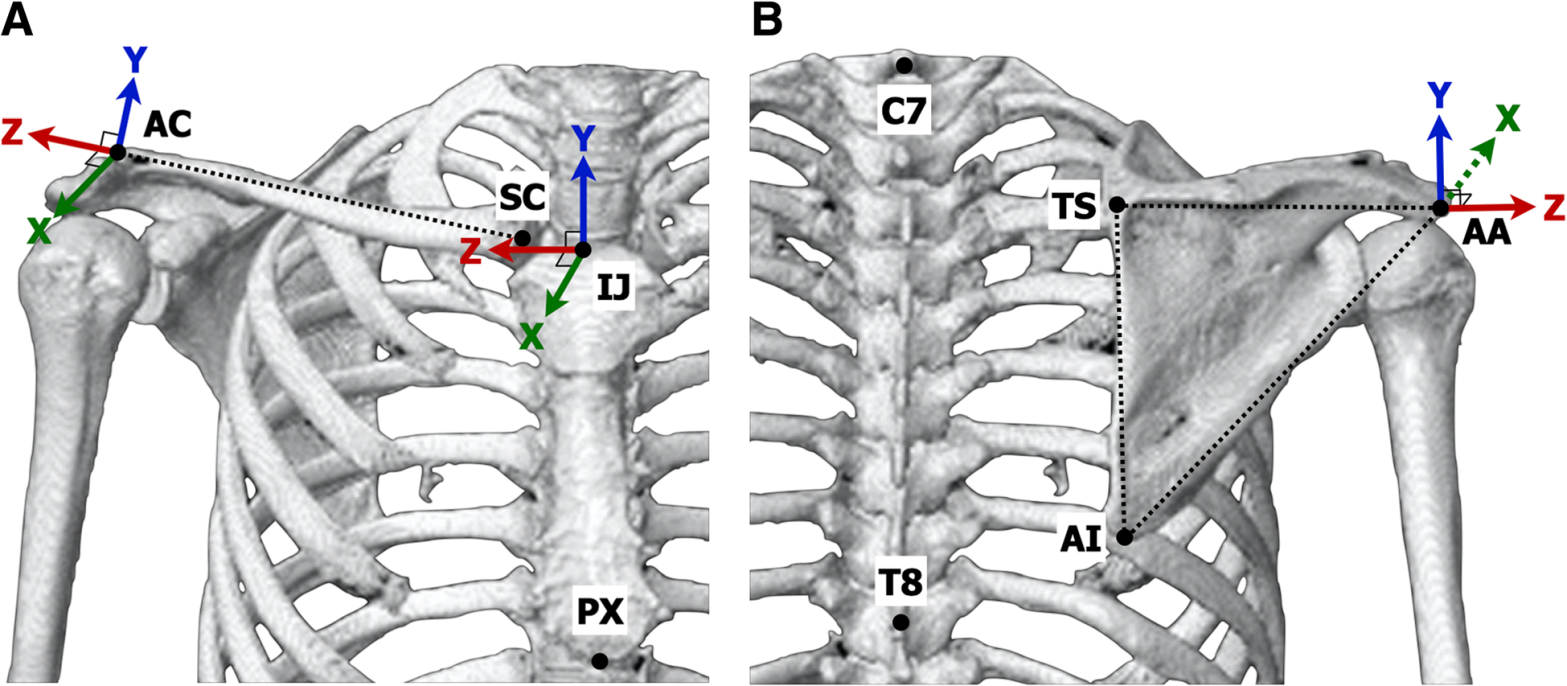
The local 3-dimensional coordinate systems of the thorax, clavicle, and scapula. **a** The thorax and clavicular coordinate systems are defined from the specific bony landmarks on CT scans, and the angular rotation of the clavicle relative to the thorax is calculated. IJ, sternal notch; PX, xiphoid process; SC, sternoclavicular joint; AC, acromioclavicular joint. **b** The scapular coordinate systems are defined from the specific bony landmarks of the scapula, and the angular rotation of the scapula relative to the thorax is calculated. C7, 7th cervical vertebra; T8, 8th thoracic vertebra; TS, root of the scapular spine; AI, inferior angle of the scapula; AA, posterolateral edge of the acromion

The local 3-dimensional coordinate systems of the thorax, clavicle, and scapula were defined from the specific bony landmarks on both supine and standing CT scans. In the thoracic coordinate system, the Y-axis was defined as the line connecting the midpoint between PX and T8 and the midpoint between IJ and C7, pointing upward; the Z-axis was the line perpendicular to the plane formed by IJ, C7, and the midpoint between PX and T8, pointing lateral; and the X-axis was the common line perpendicular to the Z- and Y-axes, pointing forward. The origin of the thoracic coordinate system was coincident with the sternal notch. In the clavicular coordinate system, the Z-axis was defined as the line connecting SC and AC, pointing lateral; the X-axis was the line perpendicular to the clavicular Z-axis and the thoracic Y-axis, pointing forward; and the Y-axis was the common line perpendicular to the X- and Z-axes, pointing upward (Fig. 2a). In the scapular coordinate system, the Z-axis was defined as the line connecting TS and AA, pointing lateral; the X-axis was the line perpendicular to the plane formed by AI, AA, and TS, pointing forward; and the Y-axis was the common line perpendicular to the X- and Z-axes, pointing upward (Fig. 2b). The angular rotation of the clavicle and scapula were calculated using Cardan or Euler angles relative to the thorax following recommendations of the International Society of Biomechanics [10]. Clavicular rotation with respect to the thorax was described as clavicular elevation/depression and retraction/protraction, and scapular rotation with respect to the thorax was described as scapular upward/downward rotation, anterior/posterior tilting, and internal/external rotation [5]. Clavicular axial rotation is usually defined as 0° on the thoracic coordinate systems and could not be evaluated in this study. To assess the movements of the clavicle and the scapula, the 3-dimensional position of the clavicle center, which was defined as the midpoint between the acromioclavicular joint and the sternoclavicular joint, and that of the scapula center, which was defined as the center of gravity of the triangle consisting of the 3 bony landmarks of the scapula, were also calculated in superior/inferior, anterior/posterior, and medial/lateral directions on the thorax coordinate system.

### Statistical analysis

Statistical analyses were performed using IBM SPSS Statistics 25.0.0.0 software (IBM Corp., Armonk, NY, USA). Intrarater and interrater reliabilities were first evaluated in 20 randomly selected cases using intraclass correlation coefficients (ICCs). Repeated measurements by 1 observer with a 3-month interval (ICC model 1, 1) and blinded measurements by 2 observers (ICC model 2, 1) were performed. After determining intra- and interrater reliabilities, analyses were performed for all shoulders by 1 observer.

The values of the angular rotations of the clavicle and the scapula and of the positions of the clavicle center and the scapula center in standing CT scans were compared with those in supine CT scans using Wilcoxon signed-rank tests. Sex differences in angular rotations and positions of the clavicle and the scapula were also assessed using Mann-Whitney *U* tests. The significance level was set at 0.05 for all analyses.

## Results

**Fig. 3:**
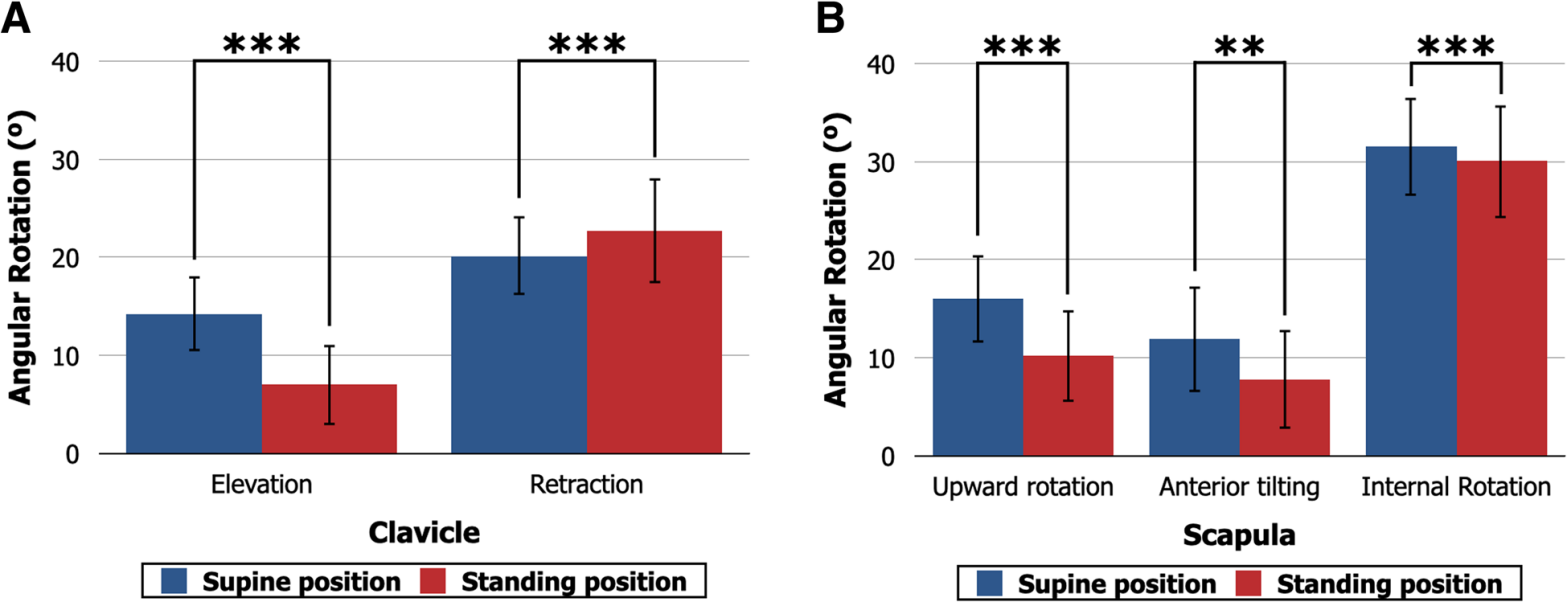
Three-dimensional angular rotations of the clavicle and scapula in the supine and standing positions. **a** Three-dimensional angular rotations of the clavicle. Compared with the supine position, the clavicle shows significantly less elevation and greater retraction in the standing position. ***P < .001. **b** Three-dimensional angular rotations of the scapula. Compared with the supine position, the scapula shows less upward rotation, anterior tilting, and internal rotation in the standing position. **P < .01. ***P < .001

Intra- and interrater reliabilities exceeded 0.8 for angular rotations (Table 1) and 0.9 for positions (Table 2) for all measurements and were regarded as excellent. Anatomical alignment of the shoulder girdle changed significantly between the supine and standing positions. In the supine position, the average clavicular angular rotation was 14° ± 4° in elevation and 20° ± 5° in retraction. Compared with the supine position, the clavicle showed significantly less elevation (7° ± 4°, *P* < .001) and greater retraction (23° ± 5°, *P* < .001) in the standing position (Fig. 3a). In the supine position, the average scapular angular rotation was 16° ± 4° in upward rotation, 12° ± 5° in anterior tilting, and 32° ± 5° in internal rotation. The scapula had significantly less upward rotation (10° ± 5°, *P* < .001), anterior tilting (8° ± 5°, *P* < .001), and internal rotation (30 ± 6°, *P* < .001) in the standing position than in the supine position (Fig. 3b). Males had significantly greater clavicular elevation and scapular anterior tilting than females (*P* ≤ .014). On the other hand, females showed greater scapular upward rotation and scapular internal rotation, and the differences became significant in scapular upward rotation in the supine position (*P* = 0.028) and scapular internal rotation in the standing position (*P* = .022) (Table 1).

**Table 1:** Three-dimensional angular rotations of the clavicle and scapula

			Mean ± SD			Sex difference	ICC (95% CI)	
			Total	Male	Female	*P* value	Intrarater	Interrater
Supine position	Clavicle	Elevation (°)	14 ± 4	15 ± 4	14 ± 4	.014*	0.998 (0.995–0.999)	0.989 (0.962–0.996)
		Retraction (°)	20 ± 5	20 ± 5	20 ± 4	.494	0.991 (0.978–0.996)	0.894 (0.562–0.965)
	Scapula	Upward rotation (°)	16 ± 4	15 ± 4	17 ± 4	.028*	0.986 (0.966–0.994)	0.990 (0.903–0.997)
		Anterior tilting (°)	12 ± 5	13 ± 6	11 ± 5	.015*	0.994 (0.986–0.998)	0.998 (0.994–0.999)
		Internal rotation (°)	32 ± 5	31 ± 5	32 ± 5	.060	0.879 (0.724–0.950)	0.827 (0.617–0.928)
Standing position	Clavicle	Elevation (°)	7 ± 4	8 ± 4	6 ± 4	< .001***	0.994 (0.984–0.997)	0.940 (0.856–0.976)
		Retraction (°)	23 ± 5	23 ± 6	23 ± 5	.840	0.995 (0.988–0.998)	0.968 (0.752–0.991)
	Scapula	Upward rotation (°)	10 ± 5	10 ± 5	11 ± 4	.052	0.980 (0.952–0.992)	0.952 (0.875–0.981)
		Anterior tilting (°)	8 ± 5	10 ± 5	7 ± 5	< .001***	0.999 (0.997–1.000)	0.958 (0.899–0.983)
		Internal rotation (°)	30 ± 6	29 ± 6	31 ± 6	.022*	0.914 (0.800–0.965)	0.936 (0.847–0.974)

*SD*, standard deviation; *ICC*, intraclass correlation coefficient; *CI*, confidence interval

*P < .05

**P < .01

*** *P* < .001

**Table 2:** Three-dimensional positions of the clavicle center and scapula center

			Mean ± SD			Sex difference	ICC (95% CI)	
			Total	Male	Female	*P* value	Intrarater	Interrater
Supine position	Clavicle	Inferior (mm)	− 23.3 ± 6.0	− 26.0 ± 5.6	− 21.4 ± 5.5	.026*	0.996 (0.989–0.998)	0.908 (0.718–0.966)
		Posterior (mm)	20.0 ± 6.5	21.5 ± 7.1	18.9 ± 5.8	< .001***	0.998 (0.994–0.999)	0.986 (0.966–0.995)
		Lateral (mm)	85.4 ± 6.5	91.3 ± 4.6	81.5 ± 4.4	< .001***	0.999 (0.999–1.000)	0.987 (0.966–0.995)
	Scapula	Inferior (mm)	4.4 ± 10.8	1.9 ± 11.4	6.0 ± 10.0	< .001***	0.977 (0.944–0.991)	0.978 (0.945–0.991)
		Posterior (mm)	74.2 ± 10.7	81.4 ± 9.8	69.4 ± 8.3	.016*	0.996 (0.989–0.998)	0.997 (0.992–0.999)
		Lateral (mm)	143.2 ± 10.6	153.4 ± 7.0	136.4 ± 6.2	< .001***	0.998 (0.994–0.999)	0.995 (0.872–0.999)
Standing position	Clavicle	Inferior (mm)	− 14.2 ± 6.1	− 16.9 ± 5.8	− 12.5 ± 5.6	.004**	0.997 (0.993–0.999)	0.978 (0.707–0.994)
		Posterior (mm)	24.0 ± 7.7	26.1 ± 9.2	22.6 ± 6.3	< .001***	0.996 (0.990–0.998)	0.967 (0.918–0.987)
		Lateral (mm)	86.7 ± 7.5	93.1 ± 4.6	82.4 ± 5.8	< .001***	0.997 (0.992–0.999)	0.958 (0.897-0.983)
	Scapula	Inferior (mm)	22.0 ± 10.6	19.2 ± 11.3	23.8 ± 9.8	< .001***	0.986 (0.967–0.995)	0.994 (0.984–0.998)
		Posterior (mm)	82.0 ± 13.5	90.9 ± 13.6	76.1 ± 9.8	.009**	0.989 (0.974–0.996)	0.995 (0.988–0.998)
		Lateral (mm)	138.5 ± 11.0	148.7 ± 7.7	131.7 ± 6.8	< .001 ***	0.998 (0.995–0.999)	0.991 (0.908–0.998)

*SD*, standard deviation; *ICC*, intraclass correlation coefficient; *CI*, confidence interval

*P < .05

**P < .01

***P < .001

**Fig. 4:**
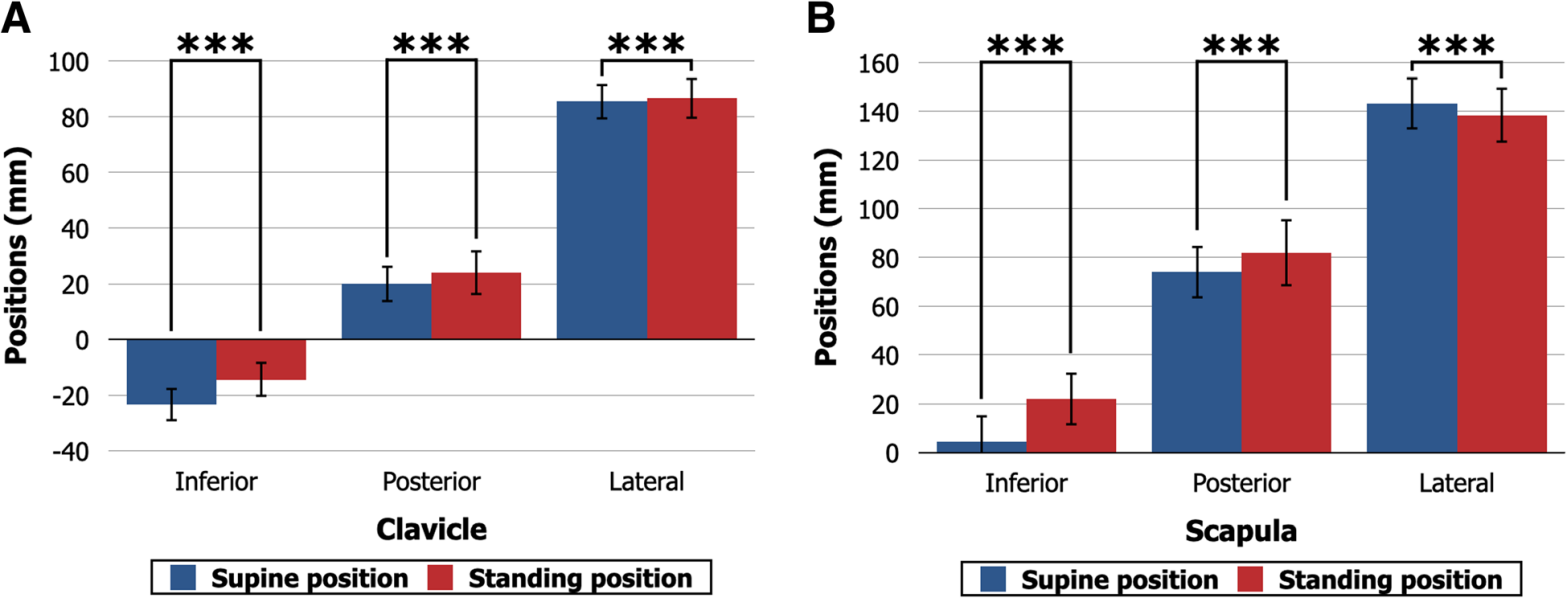
Three-dimensional positions of the clavicle and scapula in the supine and standing positions. **a** Three-dimensional positions of the clavicle center. Compared with the supine position, the clavicle center is located more inferiorly, posteriorly, and laterally in the standing position. ***P < .001. **b** Three-dimensional positions of the scapula center. Compared with the supine position, the scapula center is located more inferiorly, posteriorly, and medially in the standing position. ***P < .001

In the supine position, the clavicle center was located at 23.3 ± 6.0 mm superior, 19.9 ± 6.5 mm posterior, and 85.4 ± 6.5 mm lateral to the sternal notch. Compared with the supine position, the clavicle center was located more inferiorly (14.2 ± 6.1 mm superior, *P* < .001), posteriorly (24.0 ± 7.7 mm posterior, *P* < .001), and laterally (86.7 ± 7.5 mm lateral, *P* < .001) in the standing position (Fig. 4a). The scapula center was located at 4.2 ± 10.8 mm inferior, 74.2 ± 10.7 mm posterior, and 143.2 ± 10.6 mm lateral to the sternal notch in the supine position. Compared with the supine position, the scapula center was located more inferiorly (22.0 ± 10.6 mm inferior, *P* < .001), posteriorly (82.0 ± 13.5 mm posterior, *P* < .001), and medially (138.5 ± 11.0 mm lateral, *P* < .001) in the standing position (Fig. 4b). All of the values of the 3-dimensional positions of the clavicle center and the scapula center showed sex differences. The clavicle was located more superiorly, posteriorly, and laterally (*P* ≤ .026), and the scapula was located more superiorly, posteriorly, and laterally (*P* ≤ .016) in males than in females (Table 2).

## Discussion

Although humans spend most of their day in a standing or sitting position, little is known about the effect of gravity on the anatomical structures of the human body. This study evaluated the anatomical alignment of the shoulder girdle in the standing position using a newly developed upright CT scanning system, which had been validated to be comparable with conventional supine CT scans [7]. The present results showed that the anatomical alignment of the shoulder girdle changed significantly between the supine position and the standing position.

The present study is the first to report the normal values of the anatomical alignment of the clavicle and the scapula in the standing position using CT scans, and the values of our results were consistent with those of the past reports using electromagnetic tracking systems [3–6]. The shoulder girdle consists of the thorax, clavicle, scapula, and humerus. The scapula, in particular, is surrounded by the trapezius, rhomboid, levator scapulae, serratus anterior, deltoid, biceps, triceps, and rotator cuff muscles, [11] and scapular malposition and dyskinesis have been reported to occur with various shoulder pathologies [12–15]. Scapular posture is reported to differ between the dominant and nondominant shoulders in overhead throwing athletes [4, 6] and between throwing athletes and non-athletes, [3, 6] and the SICK (scapular malposition, inferior medial border prominence, coracoid pain and malposition, and dyskinesis of scapular movement) scapula syndrome is thought to be a cause of shoulder pain in the throwing athlete who presents with dead arm complaints [14]. Scapular malposition and dyskinesis are now judged by visual appearance, [13–15] but objective and quantitative assessment is desirable for precise assessment and adequate physical rehabilitation. Since the alignment of the clavicle and the scapula differs significantly between human positions, we believe that detailed assessment of the shoulder girdle in an upright position would help clarify the complex mechanisms and various pathologies of the shoulder girdle in future studies.

**Fig. 5:**
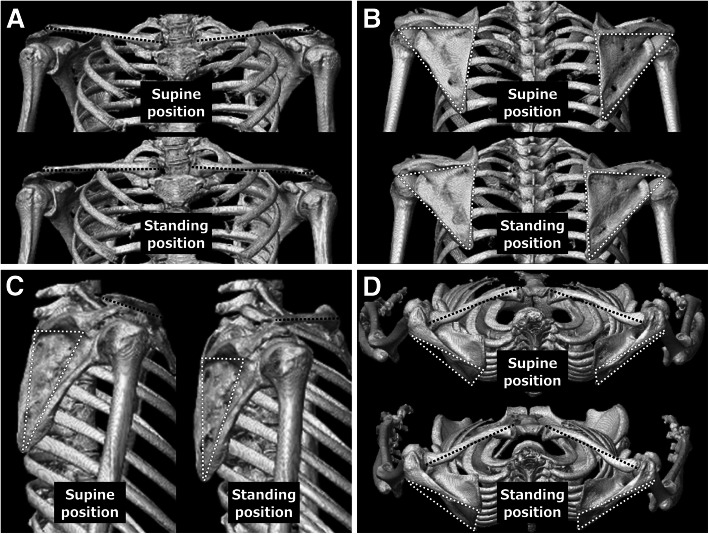
Three-dimensional CT scans in the supine and standing positions. **a** Anterior view. Compared with the supine position, elevation of the clavicle (black dotted lines) decreases in the standing position due to the effect of gravity. **b** Posterior view. Compared with the supine position, upward rotation of the scapula (surrounded by white dotted lines) decreases in the standing position due to the effect of gravity. **c** Lateral view. Compared with the supine position, the scapula (surrounded by white dotted lines) is located inferiorly and tilts posteriorly in the standing position. **d** Superior view. Compared with the supine position, the clavicle (black dotted lines) shows greater retraction, and the scapula (surrounded by white dotted lines) is located medially and rotates externally in the standing position

With the effect of gravity, the clavicle and the scapula appeared to move and rotate downwardly in the standing position compared with the supine position (Fig. 5a and b). The weight of the unilateral upper extremity corresponds to 4% to 5% of total body weight [16, 17]. Then, the inferior movement of the scapula along with the thoracic wall, which is a conical shape, is supposed to move and tilt the scapula posteriorly (Fig. 5c). We had presumed that the scapular body is close to parallel to the floor when facing upward, and the scapula has less internal rotation in the supine position compared with the standing position. Contrary to our expectation, however, the scapula rotated externally in the standing position, probably due to the shape of the thorax. In addition, the clavicular retraction was likely to increase in the standing position following the posterior translation of the acromion (Fig. 5d).

In the present study, angular rotations and positions of the shoulder girdle showed small but significant differences between males and females. Differences in body size, [18, 19] bony shape, [20], or muscles [21] between sexes are thought to cause a difference in the shoulder girdle. Since males have a larger body size than females, [18] it is natural that the distance between the sternal notch and each bone center is greater in males than in females. Nevertheless, the distance to the scapula center in the inferior direction was significantly greater in females than males, both in the supine and standing positions. There are sex differences in skeletal muscle fiber-type composition and function [21]. We assumed that the muscles anchoring the scapula to the thorax, such as trapezius, levator scapulae, and rhomboids [11], are stronger in males than females and support the scapula upwardly.

The present study had several limitations. First, the participants were healthy volunteers without any shoulder symptoms. In cases with shoulder pathology, which often occurs with dyskinesis of the shoulder girdle [13–15], alignment changes might differ from the present results. Furthermore, the angle differences found between the supine and standing positions were less than 10° and relatively small, though the present study showed alignment changes between positions. Thus, the clinical significance of the alignment changes of the shoulder girdle remained unclear. Assessment in the supine position could be another limitation of this study. For the safety of the patients, the floor of the conventional CT scanner is rounded. Although it was confirmed that no shoulders were pushed up by the floor of the CT scanner in the present study, the results for shoulder girdle alignment might differ from that in the natural supine position on a floor.

## Conclusion

Three-dimensional alignment of the shoulder girdle was evaluated in the supine and standing positions using CT scans. Due to the effect of gravity, 3-dimensional angular rotations and positions of the clavicle and scapula change significantly with position.

## Data Availability

The datasets used and/or analyzed during the current study are available from the corresponding author on reasonable request.

## Abbreviations

CT: Computed tomography
C7: The 7th cervical vertebra
T8: 8th thoracic vertebra
IJ: Sternal notch
PX: Xiphoid process
SC: The sternoclavicular joint
AC: The acromioclavicular joint
TS: The root of the scapular spine
AI: Inferior angle of the scapula
AA: Posterolateral edge of the acromion
ICC: Intraclass correlation coefficient
SICK: Scapular malposition, inferior medial border prominence, coracoid pain and malposition, and dyskinesis of scapular movement
